# *Flame Retardancy Index* for Thermoplastic Composites

**DOI:** 10.3390/polym11030407

**Published:** 2019-03-01

**Authors:** Henri Vahabi, Baljinder K. Kandola, Mohammad Reza Saeb

**Affiliations:** 1 Université de Lorraine, Laboratoire MOPS E.A. 4423, F-57070 Metz, France; 2 Institute for Materials Research and Innovation, University of Bolton, Bolton BL3 5AB, UK; B.Kandola@bolton.ac.uk; 3 Department of Resin and Additives, Institute for Color Science and Technology, P.O. Box 16765-654, Tehran, Iran

**Keywords:** *Flame Retardancy Index* (*FRI*), fire retardancy performance, thermoplastics, cone calorimetry

## Abstract

*Flame Retardancy Index*, *FRI,* was defined as a simple yet universal dimensionless criterion born out of cone calorimetry data on thermoplastic composites and then put into practice for quantifying the flame retardancy performance of different polymer composites on a set of reliable data. Four types of thermoplastic composites filled with a wide variety of flame retardant additives were chosen for making comparative evaluations regardless of the type and loading level of the additive as well as the irradiance flux. The main features of cone calorimetry including peak of Heat Release Rate (pHRR), Total Heat Release (THR), and Time-To-Ignition (TTI) served to calculate a dimensionless measure that reflects an improvement in the flame retardancy of nominated thermoplastic composites with respect to the neat thermoplastic, quantitatively. A meaningful trend was observed among well-classified ranges of *FRI* quantities calculated for the studied dataset on thermoplastic composites by which “Poor”, “Good”, and “Excellent” flame retardancy performances were explicitly defined and exhibited on logarithmic scales of *FRI* axis. The proposed index remains adaptable to thermoplastic systems whatever the polymer or additive is.

## 1. Problem Description

Additive selection for developing flame retardant systems based on thermoplastic polymers has been the subject of heated debate within the material science profession. For a given thermoplastic system, the type, loading percentage, size, shape, dispersion state, and thermal stability of flame retardant are factors responsible for the success or failure in design and implementation of a high performance system. The complexity of physical and chemical interactions between polymer chains and additives during the combustion process makes the prediction about the fire behavior of composites difficult. There is quite often a sizeable array of choices among different families of additives for applying, alone or in combination with families of identical or different nature, in a given thermoplastic system. Cone calorimetry is currently the most advanced test to capture a comprehensive image of flame retardancy performance of polymer composites [1]. Typically, peak of Heat Release Rate (pHRR), Total Heat Release (THR), and Time-To-Ignition (TTI) are the main characteristics obtained hereby. For instance, it is apparent that the lower the pHRR or THR value, the higher the fire retardancy performance of neat thermoplastic of thermoplastic composites [2,3,4]. By contrast, the more TTI, the better the performance of the system in the early stage of combustion will be [5]. Nevertheless, dissimilar origins of these measurements may bring about confusion of dominance of one criterion to another in thermoplastic systems. Therefore, those having elementary knowledge about flame retardancy, rather than professionals working in the field, may expect a criterion that considers the fingerprints of three factors (pHRR, THR, and TTI) in one. 

**Figure 1:**
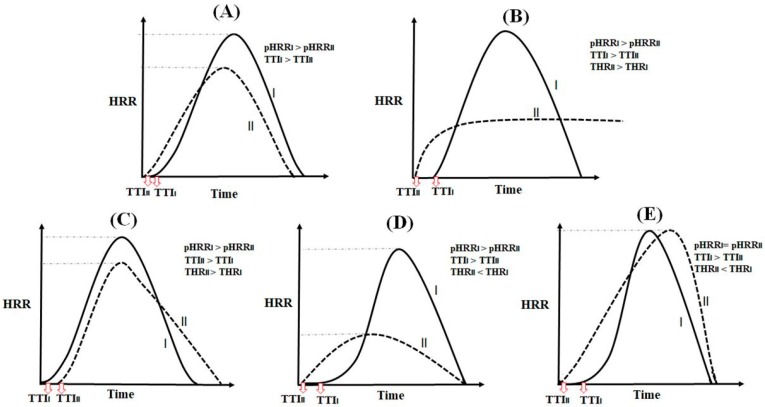
Possibilities in variation fashion of cone calorimetry HRR curves of given systems (I) and (II) that are likely to occur in different thermoplastics or in a given thermoplastic containing different FR systems (**A**–**E**) schematically patterned in this figure. Attention should be paid to the fact that such hypothetical cases are chosen among a wide variety of cases one may encounter within a conventional cone calorimetry assessment with non-interrelated variations in pHRR, THR, and TTI characteristics.

A careful survey of open literature confirms that there are several arrays of possibilities for fluctuations in pHRR, THR, and TTI values of a given thermoplastic containing different flame retardants or systems filled with one flame retardant at different levels of loading. The variation of these parameters originates from a vast variety of fire scenarios which are likely to occur in thermoplastics filled with different flame retardants each having a specified action. Some of these scenarios are schematically compared in Figure 1. The filled and dotted curves in each case among (A) to (E) scenarios in Figure 1 correspond to the fire behavior of a given thermoplastic system, whatever the amount or the type of thermoplastic polymer or flame retardant additive are. The comparison of two cone calorimetry curves for each scenario suggests that understanding and patterning the relationship between variations in pHRR, THR, and TTI parameters, even for a given system, is cumbersome. For example, the TTI value is higher for system (I) in Figure 1A compared to system (II), while the pHRR of system (I) is higher than that of system (II). It is best known that a higher TTI at the same time as a lower pHRR is desired for a higher flame retardancy performance. The question to be answered is “which characteristic among TTI or pHRR is more influential on flame retardancy performance of thermoplastic system?”. Since each characteristic has its own specific contribution to flame retardancy action, as reflected in the unit of them with “s” and “kW/m^2^” respectively assigned to TTI and pHRR, the comparison between two curves in Figure 1A for giving rank 1 and 2 to TTI or pHRR to the systems (I) and (II) in view of fire retardancy performance cannot make sense of deduction. 

The problem takes one more dimension in Figure 1B since THR enters the game. An almost different shape of cone calorimetry curves in this case remains as a signature of difficulty of judgment regarding the performance of the system against fire. There are some more possible scenarios illustrated in Figure 1C–E with their own complexities in terms of interdependence between variations in TTI and pHRR, and THR quantities, bearing in mind the fact that ultimately one system should provide the user with a higher retardancy to fire for a real-case application. For example in case (E), even if TTI_I_ is higher than TTI_II,_ the THR value is better in system (II). Moreover, even if the level of pHRR is similar in the two systems, the time to pHRR_II_ is higher than that of pHRR_I_. The aforementioned scenarios patterned in Figure 1 are examples amongst a wide variety of fire scenarios for which TTI, pHRR, and THR quantities are not alone indicative of fire retardancy character of the system or cannot in such a vague non-interrelated manner reflect flame retardancy performance of thermoplastic systems. Since TTI, pHRR, and THR have a different nature, the lack of a universal criterion for measuring good flame retardancy performance of a thermoplastic composite in the presence of different types of flame retardant systems, would cause decision-making to be very difficult.

## 2. Background and Methodology

For evaluating the flame retardancy performance of polymers, one may need to visualize the hidden phenomena behind fire scenarios. For example, THR has a unit of energy, but TTI is the time scale that demonstrates the resistance of the system against the appearance of flame at the initial stage of a fire. Therefore, they are inherently of a different nature and cannot be considered alone or in combination as a good criterion for evaluating the flame retardancy of thermoplastic composites. The other difficulty with measuring fire retardancy performance is that the situation of interaction of additives with polymers is always unknown. Hirschler [6] defined “Fire Performance Index”, FPI, in brief, as the ratio of the TTI to the pHRR having the unit of sm^2^/kW. The FPI appeared as a first-order indicator of tendency to flashover. The higher FPI values could principally specify a higher fire retardancy performance when a higher numerator, a lower denominator, or both moving in the aforementioned directions could be observed. A lower pHRR was simultaneously required for achieving higher performance levels. A wide variety of systems have been studied and concluded that such an approach would be a good measure for flame retardancy assessment. Nevertheless, one may need a simpler way to evaluate the function of flame retardants used in thermoplastic composites, such as a dimensionless criterion which could eliminate the need for simultaneous evaluation of two different measures with their own dimensions each reflecting a complexity of explanation. The new criterion had to be simple, universal, including three main parameters (pHRR, THR and TTI), and critically dimensionless to image the fingerprint of fire in a given thermoplastic composite.

To develop the idea that a universal dimensionless index is necessary, there is a perquisite to distinguish one thermoplastic composite from the other in terms of flame retardancy performance. Following the first steps taken in the aforementioned study, the plot of THR (MJ/m^2^) (Y-axis) versus pHRR/TTI (kW/m^2^.s) (X-axis) could be considered as a new pattern of fire retardancy performance. The lower X and Y axes were looked for when expecting a higher performance from a thermoplastic composite. In this sense, a huge body of literature was searched to find thermoplastic composites in which only one kind of additive was used. To give the research a versatile character, four types of polymer matrices were selected among different families of thermoplastic: polypropylene (PP) as a commodity highly flammable polymer, poly(methyl methacrylate) (PMMA) as an engineering polymer, poly(lactic acid) (PLA) as a biopolymer, and poly(ethylene-*co*-vinyl acetate) (EVA) as an emerging polymer widely used in the cable industry. Table 1 summarizes the whole data extracted from the literature on cone calorimetry features of selected systems. 

**Figure 2:**
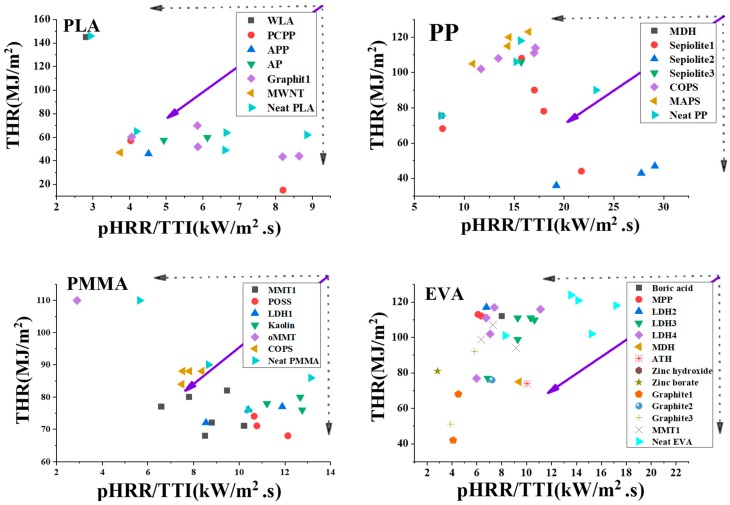
The plots of THR (MJ/m^2^) against pHRR/TTI (kW/m^2^.s) values obtained from cone calorimetry data with mauve arrows signifying improvement in flame retardancy performance for PLA, PP, PMMA, and EVA thermoplastic composites containing bewildering arrays of additives.

The variations of THR versus pHRR/TTI for the composites based on PP, PMMA, EVA, and PLA are then presented in Figure 2. This figure visualizes the actions of additives of different types and families in the aforementioned thermoplastic matrixes for evaluating the flame retardancy behavior of composites. Two points should be cared when using these plots. First, since data are picked out from different sources considering the limited access to reports in which the desired cone calorimetry data could be extracted from, each plot for the assigned thermoplastic contains several symbols denoting the mentioned neat polymer. The diversity of flame retardancy levels of neat polymers in each plot is an indication of the difference in flame retardancy of the selected polymer matrix in terms of molecular weight and viscosity obviously controlled over flame retardancy behavior of the specified thermoplastic. Second, the distribution pattern of flame retardancy of thermoplastic composites featured by THR (MJ/m^2^) and pHRR/TTI (kW/m^2^.s) in any specified case can be detected with symbols of spread positions in the area of the plot that can be noticed as a signature of complexity of the behavior of system against fire. The mauve arrows in the plots represent the direction toward which a desired flame retardancy improvement was likely to ensue. When THR and pHRR/TTI together take a low value, the desired flame retardancy will be recognized. However, the comparison is qualitative and there is no measure for quantifying the performance of systems. In other words, the unanswered question remaining with such a qualitative plot is: “Which polymer matrix or flame retardant additive would be the best choice?” The main complexity of providing an answer to the above question is that the very broad distribution of symbols (assigned to additives marked in each plot) gives a complex nature to the performance of flame retardant additives, each with its own hidden effect on the fire behavior of the system, and they cannot explicitly be held responsible for their actions. 

Here, we define and put into practice the "*Flame Retardancy Index*”, *FRI,* as a simple yet universal dimensionless index in terms of pHRR, THR, and TTI. The *FRI* was defined as the ratio of $\mathrm{THR}*\left(\frac{\mathrm{pHRR}}{\mathrm{TTI}}\right)$ between the neat polymer and the corresponding thermoplastic composite containing only one flame retardant additive:

(1) $$Flame\;Retardancy\;Index\;\left(FRI\right)=\frac{{\left[\mathrm{THR}\;*\;\left(\frac{\mathrm{pHRR}}{\mathrm{TTI}}\right)\right]}_{\mathrm{Neat}\;\mathrm{Polymer}}}{{\left[\mathrm{THR}\;*\;\left(\frac{\mathrm{pHRR}}{\mathrm{TTI}}\right)\right]}_{\mathrm{Composite}}}$$

**Figure 3:**
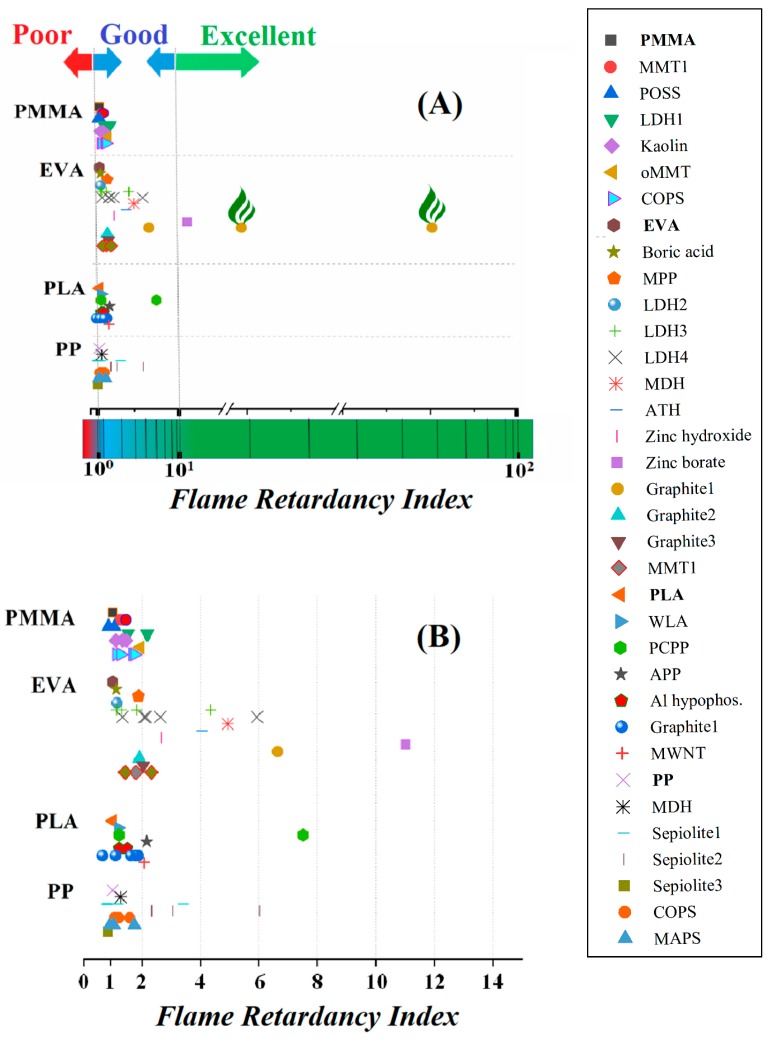
The calculated *FRI* for PLA, PP, PMMA, and EVA thermoplastic composites containing bewildering arrays of additives demonstrating the quality of flame retardancy in terms of “*Poor*”, “*Good*”, and “*Excellent*” performance: (**A**) a global view and (**B**) a closer view.

In principal, it is expected that by introducing the flame retardant additive and dividing the term calculated for the neat polymer to that of the thermoplastic composite, a dimensionless quantity greater than 1 is obtained. This operation and incorporation of a neat polymer value in the *FRI* formula lets us compare the different systems regardless of the nature of the used polymer in terms of molecular weight or viscosity. Having this in mind and by calculating *FRI* for reliable data on thermoplastic systems given in Table 1, we defined “*Poor*”, “*Good*”, and “*Excellent*” fire retardancy features assigned to well-classified ranges of *FRI* quantities colored in red, blue, and green, respectively (Figure 3). Classically saying, the quality of the flame retardancy performance can be assigned to the quantitative levels defined below in terms of ranges in *FRI* values (Figure 3). It is expected to see the value of 10^0^ from Equation (1) as the low limit for flame retardancy performance below which the addition of a flame retardant additive is not reasonable. This is representative of a system in which the addition of a flame retardant additive inversely affects performance. Therefore, *FRI* < 1 is taken as the lowest level of flame retardancy symbolized as *“Poor*” performance. Since data are gathered from a variety of reports in which different polymers (PP, PLA, PMMA, and EVA) filled with different amounts of various additives are included, the trend in the variation pattern of *FRI* can be considered as a snapshot of the behavior of thermoplastic composites when subjected to fire. From Figure 3A it can be observed that *FRI* values up to 10^1^ (1 ˂ *FRI* ˂ 10) are the most probable case, which are nominated as the *“Good*” zone colored in blue. A closer view of *“Poor*” and *“Good*” situations is provided in Figure 3B. The majority of *FRI* values calculated by Equation (1) are located in between 1 and 10. Moreover, in contrast to our initial expectation, some *FRI* values took quantities below 10^0^. This suggests that flame retardants can also contribute to combustion and, therefore, even in the presence of a flame retardant, the flame retardancy of a polymer can be worsened. The *FRI* values between 10^1^ and below 10^2^ (10 ˂ *FRI* ˂ 100) are labeled “*Excellent*” and are distinguished by a green background in Figure 3A. Three points are located in the excellent flame retardancy zone. These systems contain EVA and expanded graphite [12] or zinc borate [11]. Expanded graphite is well known as a conventional flame retardant that acts on the barrier effect of a formed char, in terms of quality and quantity, during the combustion. It can also change the thermal conductivity of a polymer. Its incorporation into polymer leads to the increase of thermal conductivity and, therefore, to the dissipation of heat at the surface of the polymer. It is worth mentioning that the loading percentage of expanded graphite is unusually and extremely high for this type of flame retardant in the aforementioned study [12]. Zinc borate is a char promoter and during the degradation, forms compact char, which protects the underlying polymer from fire. Once again, in this study, the incorporation percentage of zinc borate is higher than the usual quantity [24].

The dimensionless index nominated as *FRI* is useful for the comparative evaluation of the flame retardancy performance of thermoplastic systems regardless of the types of polymers and additives used. However, for now, this index is only adapted to simple fire scenarios where one peak of HRR appears during combustion. More complex fire scenarios can happen when two or more pHRR are compared to a second curve. In that case, one may need a high flame resistance rather than flame retardancy and, therefore, the char quantity and quality should be meticulously considered as well. 

## 3. Conclusions

Nowadays, the most important challenge in the flame retardancy field is to develop an efficient and low cost flame retardant system with non-environmental threats [25]. The evaluation of flame retardant system efficiency is a crucial step in the development of new materials. In this regard, the use of cone calorimeter data is currently well known as the best fire bench-scale method and provides useful information. However, the complexity and multitude of fire scenarios as well as the multitude of non-correlated parameters (HRR, TTI, and THR) obtained in the cone calorimeter test remains a source of error and distorted judgment in flame retardancy evaluation. The lack of a universal parameter which can quantify and allow the comparison of different flame retardant systems was pointed out by Schartel, Wilkie and Camino in 2016 [26]. This work is the first attempt to define a simple yet universal dimensionless index, hereafter known as *Flame Retardancy Index* (*FRI*), which appears informative and utilitarian for making a judgment about the effect of the performance of fillers/additives on flame retardancy behavior and properties of thermoplastics. Regardless of the irradiance flux and concentration of additives within the system, the approach is applied to a series of reliable data on PP, PMMA, EVA, and PLA composites. Surprisingly enough, a meaningful trend on a logarithmic scale was observed among well-classified ranges of *FRI* quantities calculated for the studied dataset, by which “Poor”, “Good”, and “Excellent” flame retardancy performances are explicitly defined and exhibited on the *FRI* axis for cases assigned to values below 10^0^, in between 10^0^ and 10^1^, and above 10^1^, respectively. We believe that this idea can help investigators to, in a simple manner, judge the performance of their systems when subjected to a flame; however, it must still be generalized to more cases for the sake of relevance and powerful evaluation.

## Figures and Tables

**Table 1:** Cone calorimetry data on pHRR, THR, and TTI characteristics of thermoplastic composites based on PP, PMMA, PLA, and EVA matrices components filled with a wide variety of additives. In the second column, the type and wt % of filler are typically represented as X-N denoting X type additive loaded with N wt % to the base thermoplastic.

Polymer	FR (wt %)	Irradiance (kW/m²)	TTI (s)	pHRR (kW/m²)	THR (MJ/m^2^)	Ref.
**PMMA**	-	35	21	790	76	[7]
**PMMA**	MMT- 2	35	24	725	71	[7]
**PMMA**	MMT- 4	35	20	634	72	[7]
**PMMA**	MMT- 6	35	20	579	68	[7]
**PMMA**	POSS-1	35	17	789	74	[7]
**PMMA**	POSS-3	35	17	825	68	[7]
**PMMA**	POSS-6	35	20	765	71	[7]
**PMMA**	-	50	9	1129	86	[8]
**PMMA**	LDH-3	50	10	915	77	[8]
**PMMA**	LDH-5	50	12	790	76	[8]
**PMMA**	LDH-10	50	9	615	72	[8]
**PMMA**	MMT-3	50	12	777	82	[8]
**PMMA**	MMT-5	50	13	625	80	[8]
**PMMA**	MMT-10	50	13	508	77	[8]
**PMMA**	Kaolin-3	50	10	1014	80	[8]
**PMMA**	Kaolin-5	50	10	970	76	[8]
**PMMA**	Kaolin-10	50	7	875	78	[8]
**PMMA**	-	35	69	620	110	[8]
**PMMA**	OMMT-10	35	74	320	110	[9]
**PMMA**	-	35	31	779	90	[9]
**PMMA**	Styreneoligomer-containing MMT (COPS)-2.5	35	32	737	88	[9]
**PMMA**	Styreneoligomer-containing MMT(COPS)-5	35	34	689	88	[9]
**PMMA**	Styreneoligomer-containing MMT(COPS)-15	35	39	629	84	[9]
**PMMA**	Styreneoligomer-containing MMT(COPS)-25	35	45	663	88	[9]
**EVA**	-	35	65	1680	124	[10]
**EVA**	Boric acid-10	35	35	899	112	[10]
**EVA**	Melamine polyphosphate-10	35	47	715	112	[10]
**EVA**	MgAl–LDH-10	35	33	793	117	[10]
**EVA**	-	35	58	2027	118	[11]
**EVA**	MgAl–borate LDH-3	35	35	1169	110	[11]
**EVA**	MgAl–borate LDH-5	35	36	1146	111	[11]
**EVA**	MgAl–borate LDH-10	35	36	1031	111	[11]
**EVA**	MgAl–borate LDH-20	35	40	919	99	[11]
**EVA**	MgAl–borate LDH-40	35	43	530	77	[11]
**EVA**	ZnAl–borate LDH-3	35	48	1287	116	[11]
**EVA**	ZnAl–borate LDH-5	35	51	867	117	[11]
**EVA**	ZnAl–borate LDH-10	35	53	750	111	[11]
**EVA**	ZnAl–borate LDH-20	35	38	721	102	[11]
**EVA**	ZnAl–borate LDH-40	35	51	460	77	[11]
**EVA**	MDH-40	35	63	703	75	[11]
**EVA**	ATH-40	35	54	743	74	[11]
**EVA**	Zinc hydroxide-40	35	36	1079	52	[11]
**EVA**	Zinc borate-40	35	50	231	81	[11]
**EVA**	-	35	61	1709	121	[11]
**EVA**	Melamine polyphosphate-10	35	48	689	113	[11]
**EVA**	-	35	53	836	101	[12]
**EVA**	expanded graphite-10	35	87	307	68	[12]
**EVA**	natural graphite-10	35	50	549	76	[12]
**EVA**	graphite oxide-10	35	63	536	92	[12]
**EVA**	Expanded graphite-16 (20phr)	35	186	198	51	[12]
**EVA**	Expanded graphite- 24 (30phr)	35	409	172	42	[12]
**EVA**	-	35	48	1550	102	[13]
**EVA**	MMT- 3	35	44	860	94	[13]
**EVA**	MMT- 5	35	36	780	107	[13]
**EVA**	MMT- 10	35	44	630	99	[13]
**PLA**	-	35	78	427	146	[14]
**PLA**	Aryl polyphenylphosphonate (WLA)-7	35	87	407	145	[14]
**PLA**	-	35	60	272	65	[15]
**PLA**	PCPP-10	35	54	230	57	[15]
**PLA**	PCPP-20	35	47	123	15	[15]
**PLA**	-	35	60	272	65	[16]
**PLA**	APP-15	35	70	208	46	[16]
**PLA**	-	35	57	549	62	[17]
**PLA**	Aluminum hypophosphite-10	35	45	368	60	[17]
**PLA**	Aluminum hypophosphite-20	35	41	285	57.7	[17]
**PLA**	Expanded Graphite-10	35	46	244	60.2	[17]
**PLA**	Expanded Graphite-20	35	46	356	43.5	[17]
**PLA**	-	35	88	324	49	[18]
**PLA**	MWNT-5	35	95	176	47	[18]
**PLA**	-	50	64	425	64	[19]
**PLA**	Expandable graphite-1	50	44	410	70	[19]
**PLA**	Expandable graphite-5	50	43	380	44	[19]
**PLA**	Expandable graphite-10	50	60	305	52	[19]
**PP**	-	50	37	584	75.6	[20]
**PP**	MDH-10	50	33	471	65.9	[20]
**PP**	Sepiolite-5	50	24	533	68.1	[20]
**PP**	-	35	30	2086	90	[21]
**PP**	Sepiolite- 3	35	26	1534	90	[21]
**PP**	Sepiolite- 5	35	19	1401	78	[21]
**PP**	Sepiolite- 10	35	23	957	44	[21]
**PP**	organoSepiolite- 3	35	24	1368	47	[21]
**PP**	organoSepiolite- 5	35	25	1193	43	[21]
**PP**	organoSepiolite- 10	35	24	692	36	[21]
**PP**	-	35	43	1845	118	[22]
**PP**	Styreneoligomer-containing MMT (COPS)-2.5	35	47	1953	114	[22]
**PP**	Styreneoligomer-containing MMT(COPS)-5	35	45	1889	111	[22]
**PP**	Styreneoligomer-containing MMT(COPS)-15	35	37	1448	108	[22]
**PP**	Styreneoligomer-containing MMT(COPS)-25	35	38	1191	102	[22]
**PP**	MAPS-2.5	35	44	2025	123	[22]
**PP**	MAPS-5	35	42	1738	120	[22]
**PP**	MAPS-15	35	39	1651	115	[22]
**PP**	MAPS-25	35	41	1139	105	[22]
**PP**	-	35	54	1610	106	[23]
**PP**	Sepiolite-0.5	35	48	1701	108	[23]
**PP**	Modified Sepiolite-0.5	35	46	1665	106	[23]
